# Thermodynamic and Kinetic Insights into Stop Codon Recognition by Release Factor 1

**DOI:** 10.1371/journal.pone.0094058

**Published:** 2014-04-03

**Authors:** Krista Trappl, Merrill A. Mathew, Simpson Joseph

**Affiliations:** Department of Chemistry and Biochemistry, University of California San Diego, La Jolla, California, United States of America; University of Lethbridge, Canada

## Abstract

Stop codon recognition is a crucial event during translation termination and is performed by class I release factors (RF1 and RF2 in bacterial cells). Recent crystal structures showed that stop codon recognition is achieved mainly through a network of hydrogen bonds and stacking interactions between the stop codon and conserved residues in domain II of RF1/RF2. Additionally, previous studies suggested that recognition of stop codons is coupled to proper positioning of RF1 on the ribosome, which is essential for triggering peptide release. In this study we mutated four conserved residues in *Escherichia coli* RF1 (Gln185, Arg186, Thr190, and Thr198) that are proposed to be critical for discriminating stop codons from sense codons. Our thermodynamic and kinetic analysis of these RF1 mutants showed that the mutations inhibited the binding of RF1 to the ribosome. However, the mutations in RF1 did not affect the rate of peptide release, showing that imperfect recognition of the stop codon does not affect the proper positioning of RF1 on the ribosome.

## Introduction

During translation termination, the newly synthesized protein is released from the ribosome by class I release factors (RF1 and RF2 in *E. coli*) [1], [2]. RF1 and RF2 recognize the mRNA stop codon in the ribosomal A site and catalyze peptidyl-tRNA hydrolysis. RF1 recognizes the stop codons UAA and UAG, whereas RF2 recognizes the stop codons UAA and UGA [3]. It is critical for cells that class I release factors discriminate strongly against sense codons in order to avoid premature termination. Genetic and biochemical studies indicated that a conserved “tripeptide anticodon” motif (PxT in RF1 and SPF in RF2) is essential for stop codon recognition [4]. Interestingly, RF1/RF2 with mutations in this tripeptide anticodon motif are functional *in vivo* [4]. Furthermore, mutations in RF1/RF2 that are located far from the tripeptide anticodon motif can alter the specificity of RF1/RF2 [5]–[7]. These results suggest that stop codon recognition is not entirely determined by the tripeptide anticodon motif but requires additional residues in RF1 and RF2.

**Figure 1:**
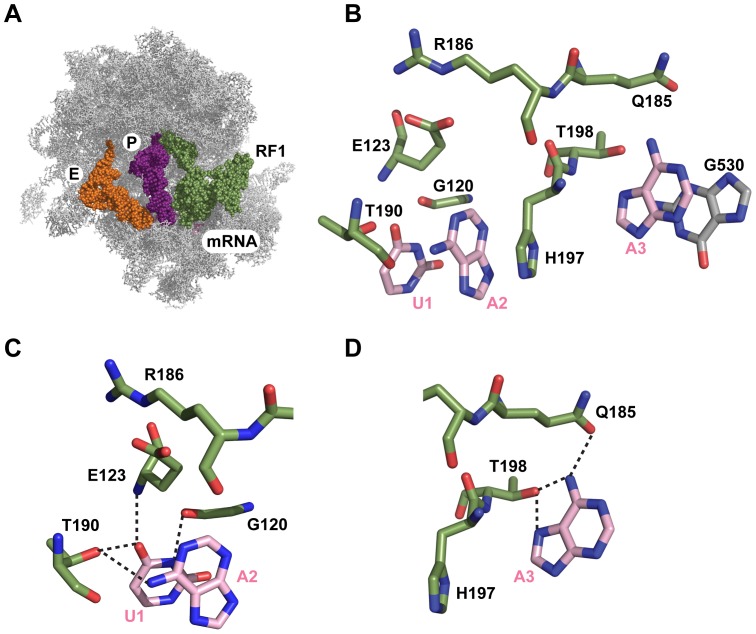
Structure of RF1 bound to the ribosome. (A) RF1 (green) bound to the ribosome (grey) in the ribosomal A site with P site tRNA (purple), E site tRNA (orange), and mRNA (pink). (B) Detailed view on the decoding site showing RF1 residues (green), base G530 of 16S rRNA (grey) and the stop codon UAA (pink). The structure figures were prepared from PDB file 3D5A using PyMol. (C) and (D) Close up of the interactions between the stop codon (pink) and the RF1 residues (green). *E. coli* numbering is used for RF1 residues. Hydrogen bonds are indicated by the dotted lines.

Recent crystal structures of RF1 and RF2 bound to the ribosome provide a structural basis for stop codon recognition (Figure 1) [8]–[11]. Consistent with genetic studies, the crystal structures showed that the tripeptide anticodon motifs of RF1/RF2 interact with the stop codons in the ribosomal A site. Importantly, several other conserved residues in RF1/RF2 also interact with the stop codons. Recognition of the stop codon is achieved through a network of hydrogen bonds and stacking interactions between the stop codon and residues in domain II of RF1/RF2 [8]–[11]. In the case of RF1, the first nucleotide of the stop codon forms hydrogen bonds with Gly120 (Gly116), Glu123 (Glu119), and Thr190 (Thr186) of RF1 (*E. coli* numbering is used throughout with *Thermus thermophilus* in brackets) [8]. The second nucleotide of the stop codon forms hydrogen bonds with Thr190 (Thr186) and forms a stacking interaction with His197 (His193) [8]. The third nucleotide of the stop codon is stacked on the universally conserved 16S rRNA base G530 and forms hydrogen bonds with Thr198 (Thr194) and Gln185 (Gln181) [8].

## Materials and Methods

### Buffers, Ribosomes, mRNA, tRNA and RF1 Preparation

The buffer used for all experiments was 20 mM Hepes-KOH (pH 7.6), 150 mM NH_4_Cl, 6 mM MgCl_2_, 4 mM β-mercatoethanol, 2 mM spermidine and 0.05 mM spermine. Tightly-coupled 70S ribosomes from *Escherichia coli* MRE 600 were prepared as published before [14]. Synthetic mRNAs were purchased from Dharamcon. mRNA with a UAA stop codon was synthesized with a 3′-amino-modifier C3 linker and mRNA with a UGA stop codon was synthesized with 3′-PT-amino-modifier C3 linker because the former linker was discontinued by the manufacturer. However, this does not change the chemical structure of the final 3′ amino linker after RNA deprotection (both are three carbon linkers) but labeling efficiency is higher with the new linker. Pyrene was covalently linked to the 3′-amino group of the mRNAs as described previously [15]. Native tRNA^fMet^ was used (Sigma) throughout this study. His-tagged *E. coli* RF1 and RF2 (referred to as wild type from here on) were purified as described in the QIAexpressionist manual (Qiagen). Proteins were quantified using the Bradford assay and stored at −80°C after flash freezing in liquid nitrogen.

### Construction of RF1 mutants

The RF1 mutants were made using QuickChange site-directed mutagenesis procedure (Stratagene). The correct clones were identified by automated DNA sequencing of the entire RF1 gene. RF1 mutants were overexpressed in *E. coli* BL21(DE3) and purified using the His-tag, as described for the wild type RF1 [13].

### Fluorescence Measurements and K_D_ Titrations

Tight-coupled ribosomes (0.25 μM final concentration) were heat activated at 42°C for 10 min then cooled to 37°C for 10 min. Pyrene labeled mRNA (0.33 μM final concentration) was added to the ribosomes and the reaction was incubated for 10 min at 37°C. Then, tRNA^fMet^ (0.5 μM final concentration) was added and the reaction was incubated for 30 min at 37°C. Equilibrium K_D_ titrations were performed by using a 160 μL fluorescence cuvette and the final concentration of the ribosome•mRNA•tRNA^fMet^ complex (release complex) was 5 nM for wild type RF1/RF2 and 50 nM for the RF1 mutants. Increasing amounts of RF1 were added to the release complex and incubated for at least 30 min at room temperature before measuring the fluorescence intensity in a fluorometer (Fluoromax-P, J.Y. Horiba Inc). The fluorescence emission scans were carried out with an excitation and emission band pass of 1 nm. Pyrene was excited at 343 nm and the emission intensity at 376 nm was measured. All experiments were repeated at least three times. Data were transformed and fit to the equilibrium K_D_ equation Y  =  m*((K+R+X)-sqrt ((K+R+X)∧2 - 4*R*X))/(2*R) using Graphpad Prism, where Y is the observed fluorescence intensity, X is the concentration of RF1 or RF2, m is the maximum fluorescence signal, K is K_D_ and R is the release complex concentration. The amount of total RF1 added to the reaction is indicated in the graphs.

### Stopped-Flow Binding Kinetics

Stopped-flow experiments were performed with a μSFM-20 (BioLogic) stopped-flow instrument at 25°C. Pyrene attached to the mRNA was excited at 343 nm with a band pass of 10 nm and emission intensity above 361 nm was collected using a longpass filter (361 AELP, Omega Optical, VT, USA). 0.25 μM release complexes were mixed with indicated amounts of RF1 and data was collected for 15 sec after mixing. The stopped flow traces were transformed and fit to the second-order rate equation Y  =  b+C1*exp(-k1*x)+C2*exp(-k2*x) using Graphpad Prism, where C1 and k1 are the amplitude and rate for phase 1 and C2 and k2 are the amplitude and rate for phase 2.

### Peptide Release Assay

Tight-coupled ribosomes (0.5 μM final concentration) were heat activated at 42°C for 10 min and subsequently cooled to 37°C for 10 min. Release complexes were formed by adding mRNA (1 μM final concentration) and incubating at 37°C for 10 min, followed by adding f-[^35^S]Met-tRNA^fMet^ (1.5 μM final concentration). The release complex was incubated at 37°C for 30 min and the excess [^35^S]-Met and other unbound reaction components were removed by ultrafiltration as described previously [13]. The final concentration of the release complex was adjusted to 0.5 μM by adding buffer. Peptide release was initiated by mixing 0.25 μM release complexes with 20 μM RF1 (both wild type and RF1 mutants) and aliquots of the reaction were taken at different time points and quenched with 25% formic acid. The samples were spotted on TLC plates and separated by electrophoresis as described previously [16]. All the experiments were independently repeated at least three times.

## Results

### K_D_ of RF1 binding to ribosome with the cognate stop codon UAA in the A site

**Figure 2:**
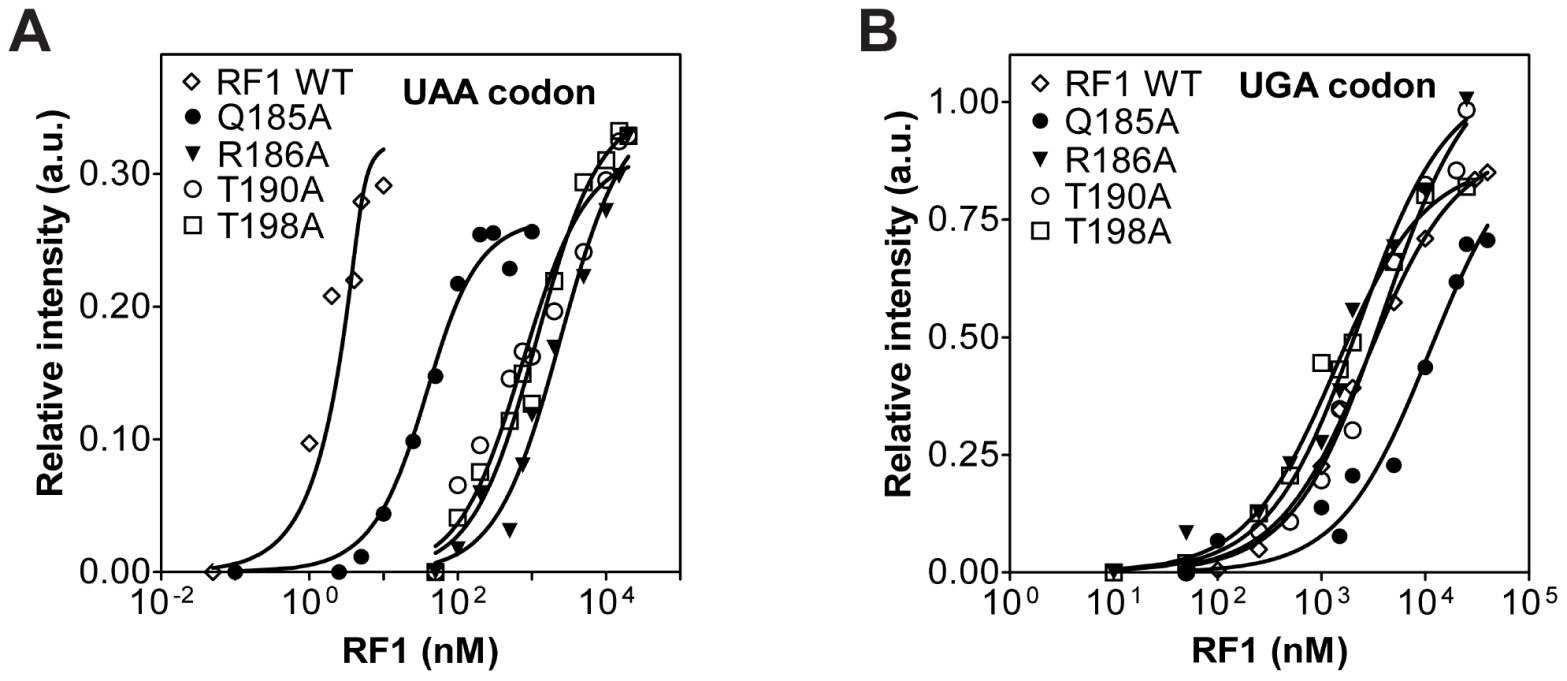
Fluorescence assay for determining the K_D_ of RF1 binding to the ribosome. (A) Changes in relative fluorescence intensity after adding increasing concentrations of wild type RF1 (open diamonds) and RF1 mutants Q185A (filled circles), R186A (filled triangles), T190A (open circles), and T198A (open squares) to ribosomes programmed with a UAA stop codon. (B) Changes in relative fluorescence intensity after adding increasing concentrations of wild type RF1 (open diamonds), and RF1 mutants Q185A (filled circles), R186A (filled triangles), T190A (open circles), and T198A (open squares) to ribosomes programmed with a UGA stop codon. Representative titration experiments without standard deviations are shown and the data were fit to the quadratic equation (black line). The total RF1 concentrations added are indicated on the x-axis.

Structural data and molecular dynamics simulation studies showed that the residues in *E. coli* RF1 that are important for stop codon recognition are Gly120, Glu123, Gln185, Arg186, Thr190, His197 and Thr198 (Figure 1B) [8], [12]. To analyze the role of these residues in RF1 for binding to the ribosome and for peptide release, we made mutants. We did not mutate Gly120 and Glu123 because the main chain carbonyl of Gly120 and the amide of Glu123 are involved in the interaction with the stop codon. A previous study analyzed the activity of His197Ala mutant [17]. We, therefore, focused our study on the remaining four key residues Gln185, Arg186, Thr190, and Thr198 in RF1 and individually changed them to an alanine by site-directed mutagenesis. Wild type RF1 and RF1 mutants were expressed, purified and analyzed for their ability to bind to ribosomes using a fluorescence-based assay that was established earlier in our lab [13]. Increasing amounts of wild type or mutant RF1 were added to a fixed concentration of 70S ribosomal complex with tRNA^fMet^ in the P site and the mRNA codon UAA in the A site. The increase in fluorescence emission intensity due to RF1 binding to the ribosome was measured for each concentration of RF1 (Figure 2A). The minimum concentration of ribosome required for the titration experiment is 5 nM for sufficient signal over noise [13], [17]. As this amount is close to the K_D_ of wild type RF1 binding to the ribosome, the K_D_ cannot be determined accurately, but it is estimated to be below 3 nM [17]. The K_D_ value is consistent with the ≈8 nM K_M_ value previously reported for wild type RF1 binding to ribosome with the UAA stop codon in the A site [18]. The RF1 mutants, overall, showed substantially higher K_D_ values compared to wild type RF1 (Figure 2A). RF1 Arg186Ala and RF1 Thr198Ala showed more than 400-fold increase (K_D_  =  1.2±0.2 μM and K_D_  =  1.3±0.2 μM, respectively), and Thr190Ala showed 250-fold increase (K_D_  =  0.8±0.1 μM), whereas RF1 Gln185Ala only showed a 20-fold increase (K_D_  =  0.060±0.02 μM) (Table 1). These results show that RF1 residues Gln185, Arg186, Thr190, and Thr198 are critical for RF1 binding to the ribosome and are in agreement with their proposed role in stop codon recognition [8] [10].

**Table 1:** Thermodynamics and kinetics of RF1 mutants binding to the ribosome.

	K_D_ UAA (μM)	K_D_ UGA (μM)	*k*_1_ UAA (μM^−1^s^−1^)
Wild type RF1	< 0.003	1.95±0.56	68±3
Gln185Ala	0.06±0.02	3.01±1.41	15±4
Arg186Ala	1.23±0.18	2.68±0.45	5±2
Thr190Ala	0.76±0.09	2.64±1.27	33±17
Thr198Ala	1.30±0.21	3.07±1.59	17±14
His197Ala*	0.35±0.03	-	71±7

*taken from 17.

### K_D_ of RF1 binding to ribosome with the non-cognate stop codon UGA in the A site

The cognate stop codons for RF1 are UAA and UAG, while UGA is the non-cognate stop codon for RF1. To investigate whether the RF1 mutants are still able to discriminate against a non-cognate stop codon, the equilibrium binding experiment was repeated using an mRNA with a UGA stop codon in the A site (Figure 2B). As expected, wild type RF1 showed strong discrimination against the UGA stop codon, reflected in the high K_D_ of 2.0±0. 6 μM. The K_D_ for the wild type RF1 binding to ribosome with the non-cognate UGA stop codon is therefore 650-fold higher compared to the cognate UAA stop codon. This discrimination was reported before [18] and was also predicted by computational studies [12]. The four RF1 mutants Gln185Ala, Arg186Ala, Thr190Ala and Thr198Ala showed 880- to 1000-fold higher K_D_ values for binding to ribosome with the non-cognate UGA stop codon compared to RF1 binding to its cognate UAA stop codon. The K_D_ for RF1 mutants Gln185Ala, Arg186Ala, Thr190Ala, and Thr198Ala are 3.0±1.4 μM, 2.7±0.5 μM, 2.6±1.3 μM, and 3.1±1.6 μM, respectively (Table 1). However, the K_D_ values for the wild type RF1 and the four RF1 mutants with the non-cognate UGA stop codon are similar suggesting that the reduced affinity for the ribosome is mainly due to the strong discrimination against the non-cognate UGA codon in the A site [18].

### Kinetics of RF1 mutants binding to the ribosome

**Figure 3:**
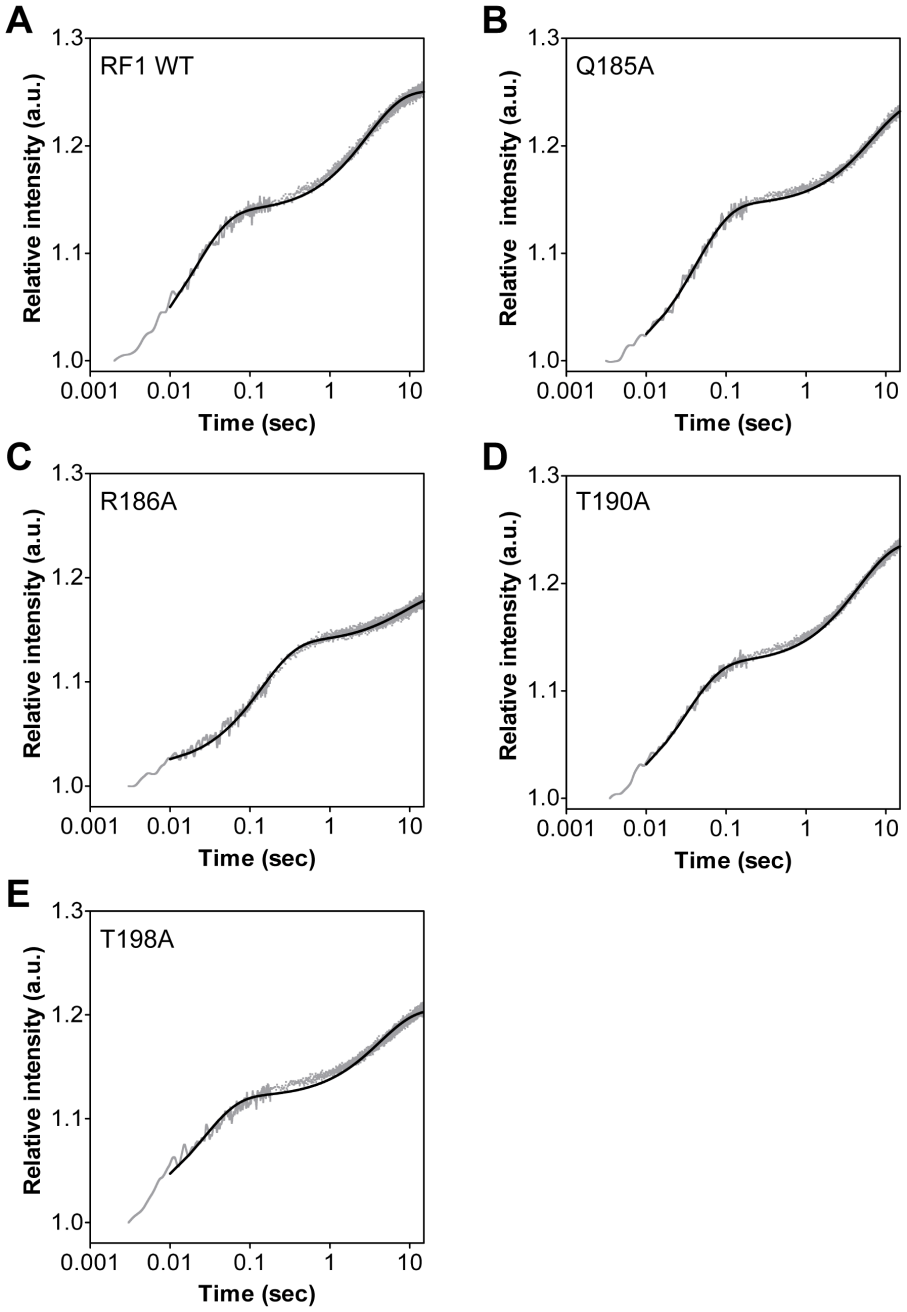
Kinetics of wild type RF1 and RF1 mutants binding to the ribosome. Representative stopped-flow time course of 1 μM wild type RF1 (A) and 1 μM RF1 mutants Q185A (B), R186A (C), T190A (D), and T198A (E) binding to ribosome. The time courses (grey trace) were transformed and fit to a double-exponential equation (black line) to determine the observed rates of RF1 binding (*k*_obs1_ and *k*_obs2_).

**Figure 4:**
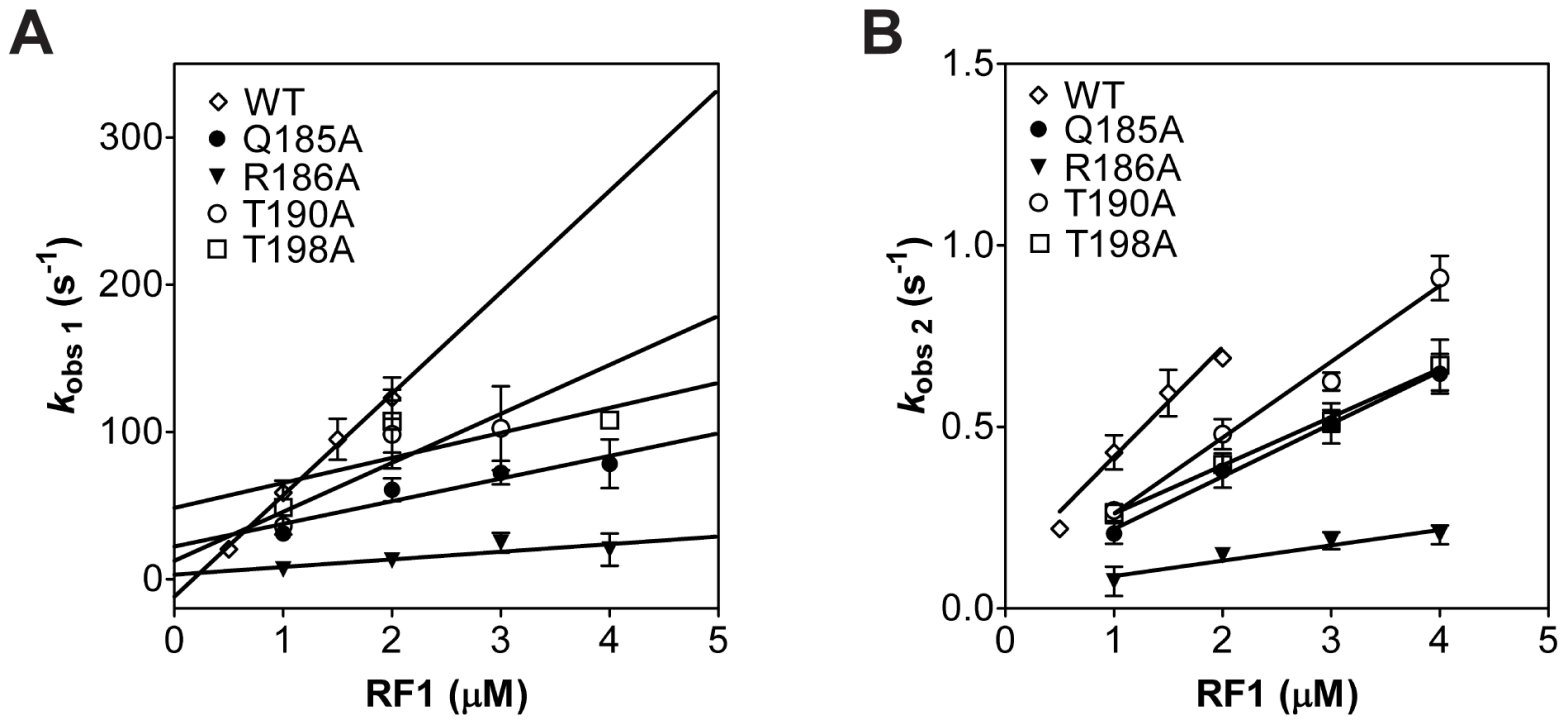
Concentration dependence of the observed rate of RF1 binding. (A) Concentration dependence of the observed rate for phase 1 of RF1 binding. Plots were fit to a linear equation to determine the association (*k*_1_) and dissociation (*k*_−1_) rate constants. (B) Concentration dependence of the observed rate for phase 2 of RF1 binding. Plots were fit to a linear equation. The standard errors from three independent experiments are shown. Indicated are wild type RF1 (open diamonds), RF1 mutants Q185A (filled circles), R186A (filled triangles), T190A (open circles), and T198A (open squares).

To determine the rate of stop codon recognition by RF1 with a cognate stop codon, transient-state kinetic studies of the various RF1 mutants were performed and compared to wild type RF1 [13], [17]. Time courses of RF1 binding to RC were determined using a stopped-flow instrument. Our previous study showed that the kinetics of RF1 binding has an initial fast phase followed by a second slower phase [17]. The amplitudes for the fast and slow phases are ≈20% and ≈10%, respectively. This biphasic increase in fluorescence could also be observed for the four RF1 mutants (Figure 3). The observed rates (*k*_obs_) of RF1 mutants binding to the ribosome were obtained by fitting the stopped-flow time courses to a double exponential equation. The experiment was performed at increasing concentrations of each RF1 mutants and a plot of RF1 concentration versus *k*_obs_ for phase 1 was used to calculate the association rate constant (*k*_1_) and the dissociation rate constant (*k*_−1_) (Figure 4A). The association rate constants of the RF1 mutants were overall 2- to 14-fold reduced compared to wild type RF1 (68 μM^−1^ s^−1^ for wild type RF1). The association rate constant for RF1 mutants Gln185Ala, Arg186Ala, Thr190Ala and Thr198Ala are 15 μM^−1^ s^−1^, 5 μM^−1^ s^−1^, 33 μM^−1^ s^−1^ and 17 μM^−1^ s^−1^, respectively (Table 1). The RF1 His197Ala mutant studied previously has an association rate constant that is nearly identical to RF1 wild type (71 μM^−1^ s^−1^) [17]. The dissociation rate constant (*k*_−1_) was determined from the y-intercept of the concentration dependence plot of phase 1. The *k*_−1_ values are 22 s^−1^, 3 s^−1^, 12 s^−1^ and 48 s^−1^ for the RF1 mutants Gln185Ala, Arg186Ala, Thr190Ala and Thr198Ala, respectively. The *k*_−1_ value for the wild type RF1 is very small and due to the magnified error of extrapolation of the data appears to be negative in the plot. The *k*_−1_ values for the RF1 mutants are slower than the previously tested RF1 His197Ala mutant (*k*_−1_ = 175 s^−1^) [17]. Thus, the association rate constants of the RF1 mutants tested in this study are affected to a greater extent than the His197Ala mutant, whereas the dissociation rate constants appear to be less affected. The second phase observed in the stopped flow time course was previously assumed to be a first order conformational change after initial binding [17]. However, the plot of RF1 concentration versus *k*_obs_ for phase 2 also showed concentration dependence and the reason for this unclear at the present time (Figure 4B). We could not determine the kinetics of RF1 mutants binding to RC with the non-cognate UGA codon because the apparent rate of association is very fast and most of the amplitude is lost in the deadtime of the instrument, which is consistent with the K_D_ values in the micromolar range.

### Kinetics of Peptide Hydrolysis by RF1 mutants

**Figure 5:**
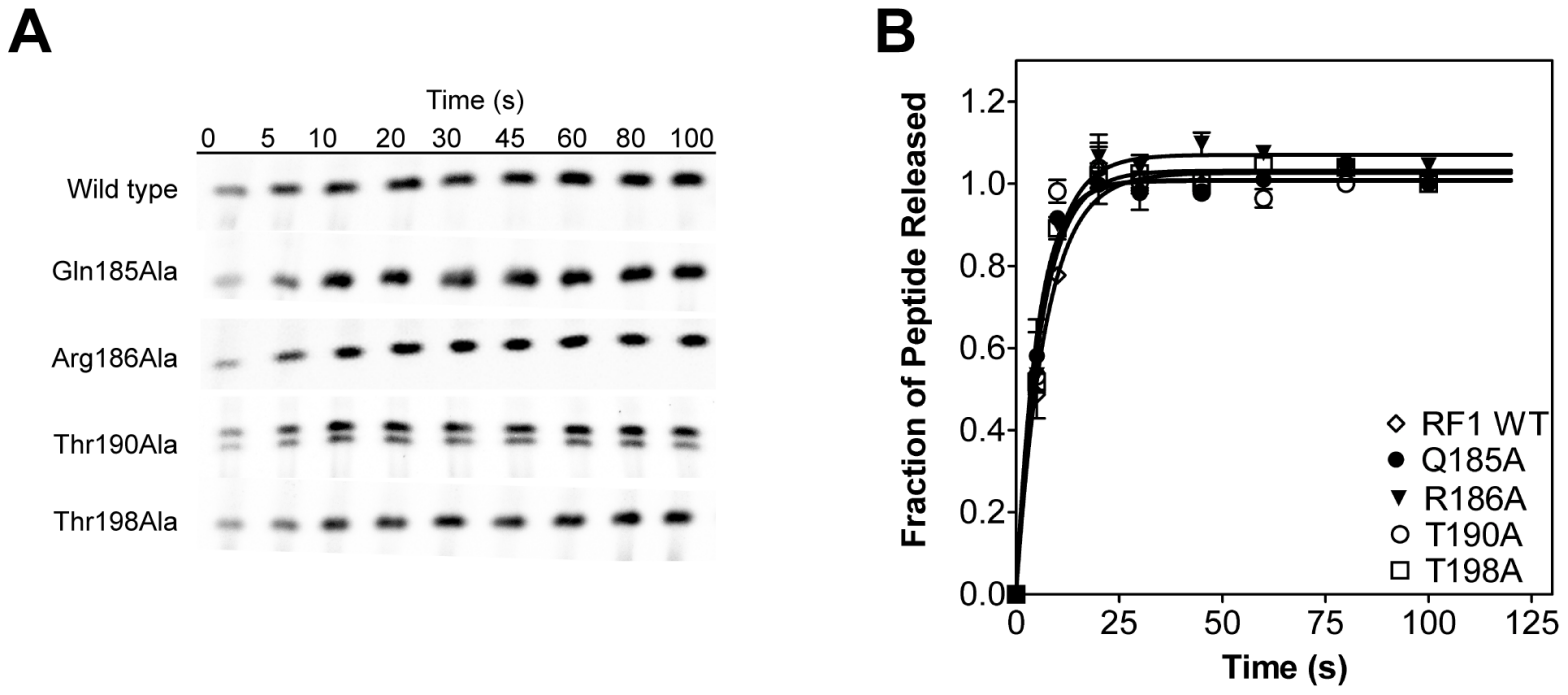
Kinetics of peptide hydrolysis by RF1. (A) Representative TLC displaying the time course of RF1-catalyzed release of [^35^S]-fMet from ribosome release complex. Labels indicate wild type RF1 and RF1 mutants. The final extent of peptide release by wild type and RF1 mutants were similar and separate filter binding studies showed that the extent of peptide release by wild type RF1 with UAA codon was >90%. (B) Graph showing the peptide release time course at saturating concentrations of wild type RF1 (open diamonds), RF1 mutants Q185A (filled circles), R186A (filled triangles), T190A (open circles), and T198A (open squares) are shown. Data were individually normalized and fit to a single-exponential equation (black line) to determine the rate of peptide release. Standard errors from at least three independent experiments are shown.

To investigate whether these mutations in RF1 affect the catalytic step, we determined the rate of peptide release. RC were formed by binding [^35^S]-fMet-tRNA^fMet^ to the ribosomal P site and peptide release time courses were performed by adding saturating amounts of RF1 (20 μM, which is at least 15-fold above the highest observed K_D_ for the RF1 mutants). RF1-catalyzed release of [^35^S]-fMet was analyzed by electrophoretic TLC and quantified with a phosphorimager (Figure 5A). To verify that saturation of RF1 binding to the ribosome was reached, the time courses were repeated with double the concentration of RF1 and identical time courses were obtained. Wild type RF1 catalyzed peptide release with a rate of 0.14±0.01 s^−1^, which agrees with data previously published [13]. The examined RF1 mutants Gln185Ala, Arg186Ala, Thr190Ala and Thr198Ala showed peptide release rates of 0.19±0.01 s^−1^, 0.16±0.01 s^−1^, 0.19±0.02 s^−1^ and 0.16±0.01 s^−1^, respectively (Figure 5B). Thus, the RF1 mutants showed no defects in peptide hydrolysis reaction rate under saturating conditions. These results suggest that the RF1 mutants are positioned correctly in the ribosome and their ability to catalyze peptide release is not compromised.

## Discussion

In this study we analyzed the role of key residues in RF1 for stop codon recognition that were identified in the crystal structures [8], [10] and by the molecular dynamics simulation study [12]. We individually changed Gln185, Arg186, Thr190, and Thr198 in RF1 to alanine and analyzed the effects of the mutations on RF1 binding to the ribosome and catalysis of peptide release. As described above, Gln185 of RF1 forms a hydrogen bond with adenine at the third position of the stop codon (Figure 1D). The observed decrease in binding affinity of the RF1 Gln185Ala mutant (20-fold higher K_D_ over wild type RF1 with the cognate stop codon) is consistent with the loss of a hydrogen bond and to some extent perturbation due to the alanine substitution in RF1. Interestingly, the RF1 mutant Gln185Ala showed an additional 50-fold higher K_D_ for binding to ribosome with the non-cognate UGA stop codon compared to the cognate UAA stop codon. This may be explained by the strong discrimination against a guanine at the second position of the stop codon by RF1 [18]. Indeed, wild type RF1 showed a 650-fold higher K_D_ for binding to ribosome with the non-cognate UGA codon compared to the cognate UAA codon. Thus, the RF1 residue Gln185 is not as important as the other residues tested for binding to the ribosome.

Interestingly, the RF1 mutant Arg186Ala showed a dramatically reduced binding affinity to ribosomes with the cognate UAA codon (400-fold higher K_D_ compared to the wild type RF1 (Table 1)). By changing Arg186 to alanine the position of Glu123 may be perturbed, which is important for recognizing uracil at the first position of the stop codon (Figure 1C). Computational study indicated that the interaction of Arg186 with Glu123 is also critical for preventing Glu123 from forming a hydrogen bond with guanine at the second position of the stop codon [12]. We found that the K_D_ for RF1 mutant Arg186Ala binding to ribosome with the non-cognate UGA codon to be about 2-fold higher compared to the cognate UAA codon. Thus, the favorable interaction that Glu123 potentially makes with the guanine at the second position cannot compensate for the unfavorable interaction that guanine makes with Thr190 in RF1. Additionally the Arg186Ala mutation showed the highest defect on the association rate constant (14-fold decreased) compared to wild type RF1, whereas it only has a modest effect on the dissociation rate of phase 1 when compared to the other RF1 mutants. These results show that mutating Arg186 in RF1, a residue that does not directly contact the stop codon, can substantially inhibit binding to the ribosome.

RF1 residue Thr190 is part of the highly conserved PxT tripeptide anticodon motif that was proposed to play a crucial role in stop codon recognition [4]. The RF1 Thr190Ala mutant showed a 250-fold higher K_D_ for binding to ribosome with the cognate UAA codon and the association rate constant was reduced by only 2-fold compared to the wild type RF1 (Table 1). Changing Thr190 to alanine disrupts two hydrogen bonds with the first and second position of the stop codon explaining the reduced binding affinity of this RF1 mutant for the ribosome.

The RF1 mutant Thr198Ala showed a 400-fold increased K_D_ for binding to RC with the cognate UAA codon but only a modest effect on the association rate constant (reduced by 4-fold compared to wild type RF1) (Table 1). Interestingly this mutant showed the most prominent effect on the dissociation rate, compared to the other RF1 mutants in our study. The Thr198Ala mutation abolishes two hydrogen bonds with the adenine at the third position explaining the reduced binding affinity of RF1 for the ribosome. Changing the codon to the non-cognate UGA stop codon reduced the binding affinity of RF1 Thr198Ala mutant additionally by about 2-fold compared to the cognate UAA stop codon. This increase in K_D_ is because guanine at the second position of the stop codon will interact unfavorably with other RF1 residues.

Our thermodynamic and kinetic analysis showed that RF1 residues Gln185, Arg186, Thr190 and Thr198 are critical for binding to the ribosome. Under physiological conditions the rate of peptide release will be dramatically reduced with the RF1 mutants because binding to the ribosome will be rate-limiting. However, under saturating conditions *in vitro*, the rates of peptide hydrolysis by the RF1 mutants were similar to the wild type RF1. This indicates that precise positioning of RF1 mutants in the ribosome is not affected, possibly because other residues in RF1 that interacts with the stop codon may be sufficient to place the GGQ motif of RF1 correctly in the peptidyl transferase center of the ribosome. In contrast, a previous study showed that RF1 is incorrectly positioned on the ribosome when a sense codon is present in the A site indicating that the final placement of RF1 is coupled to authentic stop codon recognition [19]. Additionally, we previously showed that the RF1 His197Ala mutant had a 5-fold reduced rate of peptide release compared to the wild type RF1 [17]. His197 in RF1 is unique in some respects compared to the four RF1 residues analyzed in this work because His197 does not form hydrogen bonds with the stop codons but form favorable stacking interactions with the adenine or guanine base in the second position of the stop codons (Figure 1B). The stacking interaction of His197 with the stop codon is possibly crucial for correctly positioning RF1 in the ribosome for peptide release and other residues cannot fully compensate for this special role played.
